# Derivation and validation of the ED-SAS score for very early prediction of mortality and morbidity with acute pancreatitis: a retrospective observational study

**DOI:** 10.1186/s12873-021-00410-w

**Published:** 2021-01-28

**Authors:** Joseph Miller, Yiyang Wu, Rawan Safa, Georgiana Marusca, Sandeep Bhatti, Guneet Ahluwalia, Jad Dandashi, Harold Gomez Acevedo, Naureen Farook, Ashley Scott, Vidhya Nair, Angie Adhami, Jeffrey Dueweke, Sudarshan Hebbar, Leeland Ekstrom

**Affiliations:** 1 Henry Ford Hospital, Department of Emergency Medicine, 2799 W Grand Blvd, Detroit, MI 48202 USA; 2 Wayne State University, Detroit, MI USA; 3 Nashville Biosciences, Nashville, TN USA; 4 Vanderbilt University Medical Center, Nashville, TN USA; 5 Washington University School of Medicine, St. Louis, MO USA; 6 Lake Erie College of Osteopathic Medicine, Erie, PA USA; 7 Calcimedica, La Jolla, CA USA

**Keywords:** Acute pancreatitis, Predictive score, Validation study, Emergency department

## Abstract

**Background:**

Existing scoring systems to predict mortality in acute pancreatitis may not be directly applicable to the emergency department (ED). The objective of this study was to derive and validate the ED-SAS, a simple scoring score using variables readily available in the ED to predict mortality in patients with acute pancreatitis.

**Methods:**

This retrospective observational study was performed based on patient data collected from electronic health records across 2 independent health systems; 1 was used for the derivation cohort and the other for the validation cohort. Adult patients who were eligible presented to the ED, required hospital admission, and had a confirmed diagnosis of acute pancreatitis. Patients with chronic or recurrent episodes of pancreatitis were excluded. The primary outcome was 30-day mortality. Analyses tested and derived candidate variables to establish a prediction score, which was subsequently applied to the validation cohort to assess odds ratios for the primary and secondary outcomes.

**Results:**

The derivation cohort included 599 patients, and the validation cohort 2011 patients. Thirty-day mortality was 4.2 and 3.9%, respectively. From the derivation cohort, 3 variables were established for use in the predictive scoring score: ≥2 systemic inflammatory response syndrome (SIRS) criteria, age > 60 years, and SpO2 < 96%. Summing the presence or absence of each variable yielded an ED-SAS score ranging from 0 to 3. In the validation cohort, the odds of 30-day mortality increased with each subsequent ED-SAS point: 4.4 (95% CI 1.8–10.8) for 1 point, 12.0 (95% CI 4.9–29.4) for 2 points, and 41.7 (95% CI 15.8–110.1) for 3 points (c-statistic = 0.77).

**Conclusion:**

An ED-SAS score that incorporates SpO2, age, and SIRS measurements, all of which are available in the ED, provides a rapid method for predicting 30-day mortality in acute pancreatitis.

## Background

Recently, the incidence of acute pancreatitis has risen worldwide, with results from a retrospective analysis in the US showing a 13% increase in hospital admissions over 10 years [1, 2]. The annual incidence of acute pancreatitis in the US ranged from 13 to 45 per 100,000 people [3]. While most patients who present with acute pancreatitis in the emergency department (ED) achieve positive outcomes, significant morbidity and mortality still exists [4]. On average, up to 25% will progress to severe acute pancreatitis, and pancreatic necrosis will develop in 20 to 30% of patients, both of which are associated with significant mortality [5, 6].

Multiple tools exist to aid clinicians in predicting morbidity and mortality risk associated with acute pancreatitis. These include well-known scoring systems such as Ranson’s criteria, the Acute Physiology and Chronic Health Evaluation (APACHE)-II score, and the Bedside Index for Severity in Acute Pancreatitis (BISAP) [7–9]. However, these scoring systems largely incorporate variables collected over 24 h or more of hospital admission and it may not be possible to obtain a final score until 48 h post-admission. Very early prognostic scores that apply to the ED triage and management of patients with acute pancreatitis are lacking.

The primary objective of this study was to establish a simple prognostic assessment of acute pancreatitis that clinicians can apply in the ED for early disease identification. In developing this objective, two key factors were observed that were repeatedly present early in severe acute pancreatitis. Systemic inflammatory response syndrome (SIRS) was a prominent indicator of disease severity [10–12]. Mild to moderate hypoxia was common and likely associated with early acute lung injury [13]. Hence, these findings were incorporated with other variables often available in the ED to derive and validate the ED-SAS, a simple scoring system that provides a prognostic assessment of mortality in patients admitted to the ED with acute pancreatitis.

## Methods

### Study design and setting

A retrospective analysis was performed at Vanderbilt University Medical Center (VUMC) in Nashville, TN, USA and Henry Ford Health System (HFHS) in Detroit, MI, USA. VUMC is a level 1 trauma center with approximately 125,000 annual ED visits. HFHS includes 9 EDs, 4 of which are free-standing, and 5 hospitals that experience approximately 460,000 annual ED encounters. An institutional review board at each site approved the study protocol with waiver of informed consent.

### Selection of participants

Data from VUMC were used for the derivation cohort (January 2013 to September 2017). Data from HFHS were used for the validation cohort (January 2014 to January 2018). Eligible patients were selected in similar manner to VUMC’s Synthetic Derivative, a de-identified mirror-image of the system’s electronic health records, by Nashville Biosciences, a subsidiary of VUMC established to support translational research, and from HFHS’ electronic health records (EPIC, Verona, WI). Eligible patients were 18 years of age or older, had a diagnosis of acute pancreatitis (International Classification of Diseases [ICD], 9th Revision, code 577.0 or ICD, 10th Revision, code K85), and required hospital admission within 24 h of presentation in the ED. Only the first encounter with a patient over the study assessment period was included to ensure that no patient was represented more than once in the data set. Acute pancreatitis was confirmed through elevated serum lipase levels. We excluded patients with chronic pancreatitis, hepatic failure, or with a serum lipase <3x upper limit of the normal reference range. While prognostic assessment of patients discharged from the ED may be useful, we did not include these patients, given high rates of concomitant chronic pancreatitis and reduced opportunity to obtain outcomes data given the retrospective nature of the study.

### Data collection and processing

Data collected for the derivation and validation cohorts included subject-level information on demographics; relevant comorbidities based on ICD codes; diagnoses of respiratory failure, sepsis, acute kidney injury (AKI), and other relevant sequelae as per ICD codes; mechanical ventilation determined by procedure codes for invasive mechanical ventilation; hospital procedure and visit records; mortality; and the first ED-recorded lab values and vital signs. Mechanical ventilation did not include non-invasive modes of ventilation.

Data analysis pulled electronic health data using standardized methods within each health system. For the validation cohort only, additional chart abstraction was performed to assess the accuracy of electronic data collection for the diagnosis of acute pancreatitis. Physician chart abstractors were blinded to the study hypothesis. All abstractors were trained before initiating chart abstraction and were provided with a data collection manual that included variable definitions and details. Within the validation cohort, physicians abstracted 10% of study charts to authenticate electronic data capture and to test agreement with electronic chart abstraction for the diagnosis of acute pancreatitis. We tested agreement by calculating the *K* coefficient for the diagnosis of acute pancreatitis. Complete data related to whether patients were alive or dead at the time of hospital discharge were available. For time points following hospital discharge, health system data in the validation cohort included statewide hospital mortality data.

### Outcome measures

The primary outcome was 30-day mortality. Secondary outcomes included 180-day mortality; intensive care unit (ICU) admission; length of hospital stay; presence of sepsis, respiratory failure, or AKI; and need for dialysis or mechanical ventilation. To determine these latter outcomes, we used ICD codes documented during the patient’s index hospitalization for acute pancreatitis.

### Primary data analysis

We selected predictor variables known to be associated with mortality from acute pancreatitis based on existing disease models that were present early in a patient’s ED evaluation [8, 10, 14, 15]. These predictor variables include age > 60 years, gender, self-reported race/ethnicity, the Charlson comorbidity index [16], the presence of ≥2 SIRS criteria (heart rate > 90/min, respiratory rate > 20/min, temperature > 38.0 °C or < 36.0 °C, and white blood cells > 12 × 10^9^/L or < 4 × 10^9^/L), the presence of SpO2 < 96%, and hematocrit > 44%. The SpO2 cut-off of < 96% was used as it includes the descending portion of the oxygen saturation curve. The selection of variables to include in our study’s scoring system was refined to ensure that only those that are readily available during routine ED evaluation were included.

For the derivation cohort, these variables were analyzed for their association with 30-day mortality via univariate analysis. We retained variables for the final model with *p*-values < 0.10. A complete case analysis was also performed. To reduce bias in parameter estimates, given the low event rate, multivariable logistic regression with Firth’s penalized likelihood estimate was utilized [17]. To determine the most parsimonious predictor model, the Akaike Information Criterion was assessed. In addition, model calibration using graphical assessment by loess smoothers [18] and model discrimination with area under the receiver operating characteristic curve (*c* statistic) were evaluated.

We performed logistic regression for the ED-SAS score categories with 0 as the reference to assess the odds of death and all secondary outcomes. We first performed this analysis within the derivation data set and applied this derived predictor score to the validation sample to assess its association with primary and secondary outcomes. No model recalibration was performed within the validation data. All analyses were performed with SAS 9.4 (Cary, NC). Analyses were performed between pairs of physician raters, and kappa coefficients (*K*) were calculated to determine interrater reliability.

## Results

The derivation cohort included 599 patients, and the validation cohort included 2011 patients. Interrater reliability was excellent for chart review for the diagnosis of acute pancreatitis with a *K* coefficient of 0.94. In general, mean age, SpO2, and respiratory rate were similar between the two cohorts (Table 1). The 30-day mortality rate was similar across the two cohorts with 25 (4.2%) deaths in the derivation cohort and 78 (3.9%) in the validation cohort (Table 2). Respiratory failure rates were also similar between the two cohorts (4.2% vs 5.4%, respectively), but rates of sepsis were higher in the derivation cohort than in the validation cohort (13.4% vs. 6.6%, respectively; Table 2). In addition, length of hospital stay and ICU admissions were higher in the derivation cohort (Table 2).

**Table 1:** Baseline characteristics of patients with acute pancreatitis admitted to the ED ^a^

	Derivation Cohort *N* = 599	Validation Cohort *N* = 2011
**Gender**, n (%)		
Male	316 (52.8)	977 (48.6)
Female	283 (47.3)	1034 (51.4)
**Mean Age**, years (SD)	53.4 (17.4)	56.1 (17.8)
**Race**, n (%)		
Black	81 (13.5)	487 (24.2)
White	501 (83.6)	1359 (67.5)
Other	16 (2.7)	165 (8.2)
**Ethnicity**, n (%)		
Hispanic	548 (6.2)	79 (3.9)
Non-Hispanic	562 (93.8)	1886 (93.8)
Unknown	54 (9.0)	20 (1.0)
**Vitals**, mean (SD)		
Heart Rate, bpm	91.2 (20.1)	87.9 (20.2)
Respiratory Rate, breath/min	18.4 (2.9)	18.5 (5.1)
Temperature, °C	36.7 (0.6)	36.6 (0.6)
SpO2, %	97.5 (2.7)	97.4 (2.5)
**Comorbidities**, n (%)		
Cancer	128 (27.83)	171 (13.96)
Stroke	85 (18.48)	120 (9.8)
Congestive Heart Failure	110 (23.91)	250 (20.41)
Dementia	5 (1.09)	90 (7.35)
Diabetes Mellitus	151 (32.83)	443 (36.16)
Myocardial Infarction	54 (11.74)	185 (15.1)
Chronic Kidney Disease	112 (24.35)	323 (26.37)
COPD	138 (30.00)	463 (37.8)
**Additional Predictive Variables**		
Charlson comorbidity index, mean (SD)	2.6 (2.3)	3.3 (3.7)
≥2 SIRS criteria, n (%)^b^	182 (30.4)	512 (25.5)
SpO2 < 96%, n (%)	96 (16.0)	336 (16.7)
WBC > 12 × 10^9^/L, n (%)	259 (43.2)	740 (36.8)
Hematocrit > 44%, n (%)	108 (18.0)	601 (29.9)

^a^Additional parameters were not available in the data set; ^b^Presence of 2 or more of the following: fever > 38.0 °C or hypothermia < 36.0 °C, tachycardia > 90 beats/minute, tachypnea > 20 breaths/minute, leukocytosis > 12*10 [9]/L, or leucopoenia < 4*10 [9]/L.

*COPD* chronic obstructive pulmonary disease; *SIRS* systemic inflammatory response syndrome; *WBC* white blood cells

**Table 2:** Primary and secondary outcomes upon review of electronic health record data

Outcome, n (%)	Derivation Cohort ***N*** = 599	Validation Cohort ***N*** = 2011
**30-Day Mortality**	25 (4.2)	78 (3.9)
**180-Day Mortality**	91 (15.19)	243 (12.1)
**Sepsis**	80 (13.36)	133 (6.61)
**Acute Kidney Injury**	155 (25.88)	445 (22.13)
**Respiratory Failure**	25 (4.17)	108 (5.37)
**Mechanical Ventilation**	7 (1.17)	75 (3.7)
**Dialysis**	9 (1.50)	58 (2.88)
**ICU Admission**	83 (13.86)	159 (7.91)
**Length of stay, mean days (SD)**	6.7 (7.6)	3.6 (4.8)

*ICU* Intensive care unit

**Fig. 1:**
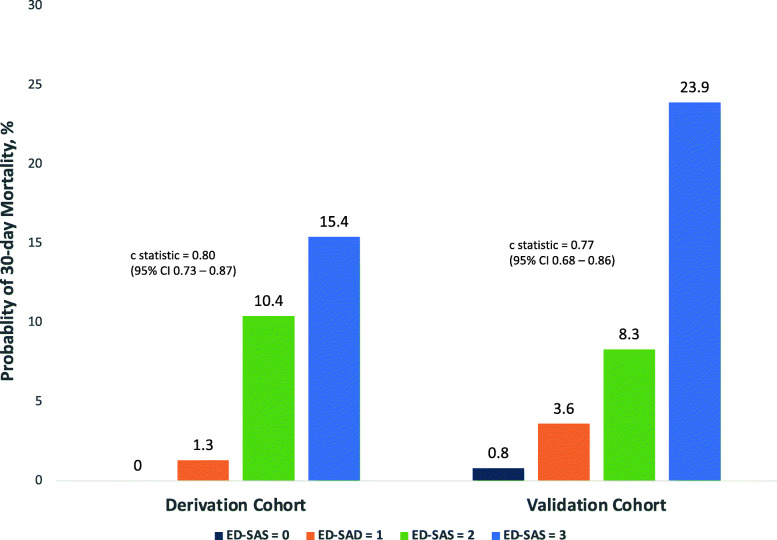
Probability of 30-day mortality based on ED-SAS scores. After application of the ED-SAS score, the probability of 30-day mortality increased incrementally with higher ED-SAS scores for both cohorts

While four variables were associated with 30-day mortality via univariate analysis in the derivation cohort, only the presence of ≥2 SIRS criteria, an ED SpO2 saturation < 96%, and age > 60 years were significant upon multivariate analysis and subsequently retained in the final ED-SAS Score (Table 3). Based on this, the final three variables included in the model were SpO2 < 96%, age > 60 years, and ≥ 2 SIRS criteria (Table 4). Each variable was equally weighed and contributed 0 or 1 points to an overall score based on the presence or absence of these three variables, resulting in an ED-SAS range of 0 to 3 points. The probability of 30-day mortality increased incrementally with higher ED-SAS scores for both cohorts (Fig. 1). In addition, increases in the odds ratio (OR) for 30-day mortality, 180-day mortality, AKI, respiratory failure, sepsis, and ICU admission were directly associated with increased ED-SAS score in the validation cohort (Table 5).

**Table 3:** Univariate analysis of acute pancreatitis predictor variables

Predictor	30-Day Mortality Odds Ratio (95% CI)
**Female gender**	1.1 (0.5–2.7)
**Age > 60 years**^a^	7.6 (2.5–23.2)
**Black Race**	0.3 (0.0–2.4)
**Hematocrit > 44%**	0.5 (0.1–2.2)
**SpO2 < 96%**^a^	5.7 (2.3–14.2)
**≥2 SIRS criteria**^a,b^	4.3 (1.0–18.7)
**Charlson Index**	1.2 (1.0–1.5)

^a^Statistically significant predictors upon implementation of the multivariant analysis that were retained in final model; ^b^Fever > 38.0 °C or hypothermia < 36.0 °C, tachycardia > 90 beats/minute, tachypnea > 20 breaths/minute, leukocytosis > 12*10 [9]/L, or leucopoenia < 4*10 [9]/L.

*SIRS* Systemic inflammatory response syndrome

**Table 4:** ED-SAS Score. A scoring system that utilizes variables readily available in the ED

Parameter	Score
**SpO2 < 96%**	1
**Age > 60 years**	1
**≥2 SIRS criteria**^a^	1
**Total Score, range**	0–3

^a^Presence of 2 or more of the following: fever > 38.0 °C or hypothermia < 36.0 °C, tachycardia > 90 beats/minute, tachypnea > 20 breaths/minute, Leukocytosis > 12*10 [9]/L, or leucopoenia < 4*10 [9]/L.

*ED-SAS* Emergency Department SpO2, Age, and SIRS, *SIRS* Systemic inflammatory response syndrome

**Table 5:** Odds of primary and secondary outcomes in validation cohort based on ED-SAS score

ED-SAS score, OR (95% CI)^a^	ED-SAS = 1	ED-SAS = 2	ED-SAS = 3
**30-day Mortality**	4.4 (1.8–10.8)	12.0 (4.9–29.4)	41.7 (15.8–110.1)
**180-day Mortality**	3.1 (1.8–5.4)	6.1 (3.4–10.9)	14.8 (7.3–30.1)
**Acute kidney injury**	2.0 (1.6–2.6)	3.3 (2.4–4.5)	5.6 (3.4–9.3)
**Respiratory failure**	2.9 (1.6–4.9)	5.7 (3.1–10.3)	7.6 (3.3–17.2)
**Sepsis**	2.9 (1.7–5.0)	7.2 (4.1–12.7)	17.1 (8.5–34.3)
**ICU Admission**	2.3 (1.5–3.5)	3.1 (1.9–5.0)	4.6 (2.3–9.3)

^a^Reference is ED-SAS of 0

## Discussion

The incidence of acute pancreatitis has been increasing worldwide. While multiple risk stratification tools exist, they come with limitations [1, 2, 7–9]. The 3 most commonly used risk stratification tools in the ED include the BISAP score, the APACHE II Scoring System, and Ranson’s criteria [1]. Imaging-based scores also exist, which grade computed tomography findings of pancreatitis, but these may not be performed routinely and may underestimate severity in patients who present early in the disease course [14, 19–21]. While these scoring systems are regularly used to predict mortality, they were not designed specifically for utility in the ED. [1, 7–9]

The ability to identify patients with acute pancreatitis who are at high-risk for mortality using a stratification tool focused on variables that are collected early in the ED could potentially lead to rapid identification of patients with poor prognoses while at the same time, identifying those patients who do not require agreement management and for whom home-based care may be more appropriate. In this study across two health systems and multiple EDs, we derived and validated the ED-SAS, a simple prognostic tool that focuses on 3 variables, all of which can be collected in the ED and are readily available in the early evaluation of a patient with acute pancreatitis: age, pulse oximetry reading on room air (SpO2 saturation < 96%), and the presence of SIRS criteria. The score was named ED-SAS as SpO2, Age, and SIRS are all variables assessed in the ED. Results from this analysis suggest increased risk of 30-day mortality with increasing ED-SAS score; ED-SAS scoring could aid in ED prognostication and influence triaging decisions.

The inclusion of advanced age, SIRS criteria, and low pulse oximetry are consistent with the existing body of literature. Age has been validated in existing inpatient prediction scores. SIRS is known to impact the clinical course and outcome in acute pancreatitis [10, 22, 23]. SIRS is also an early predictor of severe acute pancreatitis, with studies demonstrating that the severity of acute pancreatitis is greater among patients with SIRS on day 1 [24]. While less recognized than SIRS, moderate to severe arterial hypoxia is common in patients with acute pancreatitis and is related to the respiratory injury often associated with the inflammatory response seen with acute pancreatitis [10, 13]. Among patients that die early from acute pancreatitis, respiratory injury is often a prominent finding and is present in approximately 60% of all elderly patients who pass from acute pancreatitis within the first week [15, 25]. Although prior studies used arterial blood gas measurements to assess hypoxia, we relied on pulse oximetry in this study, given the infrequent use of arterial blood gas in the ED for these patients [13].

This retrospective, observational, chart review study, which relied on commonly documented predictor variables, comes with some limitations. While standardized methods for data collection were used, there remains a potential for bias, and there may be important predictors of morbidity and mortality that were not considered. Further, selection bias may exist, as patients who were discharged directly from the ED were excluded from the analysis; this limits the analysis to those with more severe cases of acute pancreatitis. We did not perform a direct comparison between the ED-SAS score and other common scoring systems or ED predictors of mortality such as the quick Sepsis Related Organ Failure Assessment score, which may have utility in evaluating acute pancreatitis, as it does in sepsis [26]. In addition, the design for assessing mortality may underestimate the true rates, given that subjects were not directly contacted, as electronic health record data were used to determine 30- and 180-day mortality. While it is possible that some deaths were not accounted for, the potential of systematic bias related to these missing data is limited by inclusion of statewide hospital death data within the validation cohort. Future research in this area should also account for additional biomarkers and clinical findings, including lactate dehydrogenase, aspartate aminotransferase, pleural effusion, and mental status. Due to the retrospective nature of data collection, we were surprised to find significant missing data for some of these variables. The ED-SAS model provides a simple assessment of high-risk patients with acute pancreatitis early in their ED course, but it was not designed to assess outcomes, the intensity of inpatient services that patients require to adequately determine eligibility for ED discharge, or outpatient management. However, as this information would be of great interest to emergency physicians, additional studies to assess this would be of value. Finally, we did not compare the ED-SAS score to clinical gestalt and are therefore unable to assess whether it provides added value to the clinical judgement of an experienced physician.

Future studies of the ED-SAS should include analysis of all patients admitted to the ED for acute pancreatitis, regardless of severity and admission to hospital. In doing so, it may be possible to utilize this predictive scoring system in a broader range of patients with acute pancreatitis, thereby mitigating selection bias.

## Conclusions

In this retrospective, observational analysis of patients hospitalized for acute pancreatitis, the ED-SAS was derived and validated as a simple predictive tool that could be used to estimate the 30-day risk of mortality using patient characteristics commonly available in the ED. This tool has the potential to aid clinicians in prognosticating patients with acute pancreatitis.

## Data Availability

Data are available on reasonable request from the corresponding author.

## Abbreviations

AKI: Acute kidney injury
APACHE-II: Acute Physiology and Chronic Health Evaluation II score
BISAP: Bedside Index for Severity in Acute Pancreatitis
ED: Emergency department
ED-SAS: Emergency department SpO2, age, and SIRS
HFHS: Henry Ford Hospital System
ICD: International Classification of Diseases
ICU: Intensive care unit
SIRS: Systemic inflammatory response syndrome
VUMC: Vanderbilt University Medical Center
